# Insulin-like Growth Factor 1 (IGF-1) measurements recorded in the OZGROW database and their relation to growth response in children currently being treated with growth hormone

**DOI:** 10.1186/1687-9856-2015-S1-P38

**Published:** 2015-04-28

**Authors:** Ian Hughes, Mark Harris, Andrew Cotterill

**Affiliations:** 1 Mater Research Institute, The University of Queensland, South Brisbane, QLD, Australia; 2 Mater Children's Hospital, South Brisbane, QLD, Australia

## Aims

IGF-1 is produced in a GH dependent fashion and measurement of IGF-1 has become increasingly common for both diagnosis of GH deficiency and to guide GH therapy. The OZGROW database records the results of IGF-1 tests performed on patients receiving GH or being assessed for GH treatment. The OZGROW database was used to determine the extent to which IGF-1 testing is performed in this population and to assess the relationship between IGF-1 levels and growth rate.

## Methods

Records were obtained for children entering the OZGROW database since 2009 and who were currently receiving GH. Number of patients, visits, and frequency of IGF-1 tests was determined. The nature of IGF-1 tests, such as the units used and whether a reference range was provided was noted. Patients with at least one IGF-1 test result were selected for further analysis. IGF-1 results were standardized (where a reference range was available) by expressing them as a % of the reference range. x% = ((x -L)/(U - L)), where x is the test result and U and L are the upper and lower values of the reference range. Growth was measured as either growth velocity (GV, cm/year) or height SDS/Year over either a 6month (+/- 10 weeks but adjusted to 6m) or 1 year (+/-13weeks adjusted to 1y). Regressions (coefficient=b) and correlations (r) were performed.

## Results

829 patients were assessed representing 7573 clinic visits. 577 (69%) did not have any IGF-1 tests recorded. 25% had one test recorded, 4% had two tests, and 1% had three tests. Another 21 IGF-1 tests were noted but not used in analyses as no reference range was given. Units used were nmol/L, mmol/L, U/ml, ug/L, mg/l, and “other”. 11 results had a reference range but no units. Overall a moderate but significant relationship was found between IGF-1% and growth: 6month GV- b=0.014cm/y/%IGF-1, r=0.16, P=0.01; 6month SDS/y- b=0.005dSDS/y/%IGF-1, r=0.31, P<0.001. 1Y GV- b=0.010, r=0.14, P=0.03; 1Y SDS/y- b=0.003, r=0.31, P<0.001. More detailed analyses stratified by indication and gender will be presented.

## Discussion and conclusions

IGF-1 has been recommended to titrate GH dose. We found significant variation between IGF-1% and growth response although the correlation was significant. There was also great variation in measurement units used and references ranges stated. Further analyses will focus on the relationship between GH dose and IGF-1% to help fully elucidate the value of IGF-1 testing in GH treatment.

